# iSoybean: A database for the mutational fingerprints of soybean

**DOI:** 10.1111/pbi.13844

**Published:** 2022-06-11

**Authors:** Mengzhu Zhang, Xiyu Zhang, Xinyu Jiang, Lei Qiu, Guanghong Jia, Longfei Wang, Wenxue Ye, Qingxin Song

**Affiliations:** ^1^ Stake Key Laboratory of Crop Genetics and Germplasm Enhancement, National Center for Soybean Improvement, Jiangsu Collaborative Innovation Center for Modern Crop Production Nanjing Agricultural University Nanjing Jiangsu China; ^2^ Jiamusi Branch of Heilongjiang Academy of Agricultural Sciences Jiamusi Heilongjiang China

**Keywords:** soybean, EMS, mutation, whole‐genome sequencing

Soybean (*Glycine max* L. Merrill) is one of the most important commercial crops worldwide. However, soybean has undergone severe genetic bottlenecks during domestication (Hyten *et al.,* 2006). It is essential to exploit novel sources of genetic diversity and to expand gene pools for soybean improvement. Plant mutation breeding has been widely used by plant breeders to create novel genetic diversity. Ethyl methanesulfonate (EMS) is a chemical mutagen believed to mainly induce point mutations, which is commonly used to develop mutant populations in soybean (Li *et al.,* 2017; Tsuda *et al.,* 2015). However, lack of genome‐wide characterization of mutations restricts the utilization of these mutant populations in the soybean community.

To provide novel genetic diversity for soybean breeding, we developed an EMS‐induced mutant population and performed whole‐genome sequencing (WGS) of 1044 mutant lines for the characterization of induced mutations (Figure 1a). About 21.5% of plants showed visual phenotypic variation in the M2 population, including leaf morphology, plant architecture and seed shape (Figure S1). On average, 76 million reads (11.4 Gb) were generated for each mutant line, resulting in an average sequencing depth of 11.2x (Table S1). In total, 6 774 731 mutations including 3 141 030 homozygous and 3 633 701 heterozygous mutations were pinpointed in 1044 mutant lines, giving an average mutation density of ~1 mutation per 150 kb for each mutant line (~6.7 mutations per kb for 1044 mutant lines) (Figure 1b, Table S1). EMS primarily induces GC > AT transitions. Totally, 4 801 170 GC > AT mutations (71% of total mutations) were detected in EMS‐treated mutant population (Table S1). To examine the error rate for mutation identification, we randomly selected 105 GC > AT and 45 non‐GC > AT mutations for validation using Sanger sequencing (Table S2). Among them, 104 GC > AT (99%) and 43 non‐GC > AT (96%) mutations were confirmed to be positive, suggesting low error rate for identification of both GC > AT and non‐GC > AT mutations in this study.

**Figure 1:**
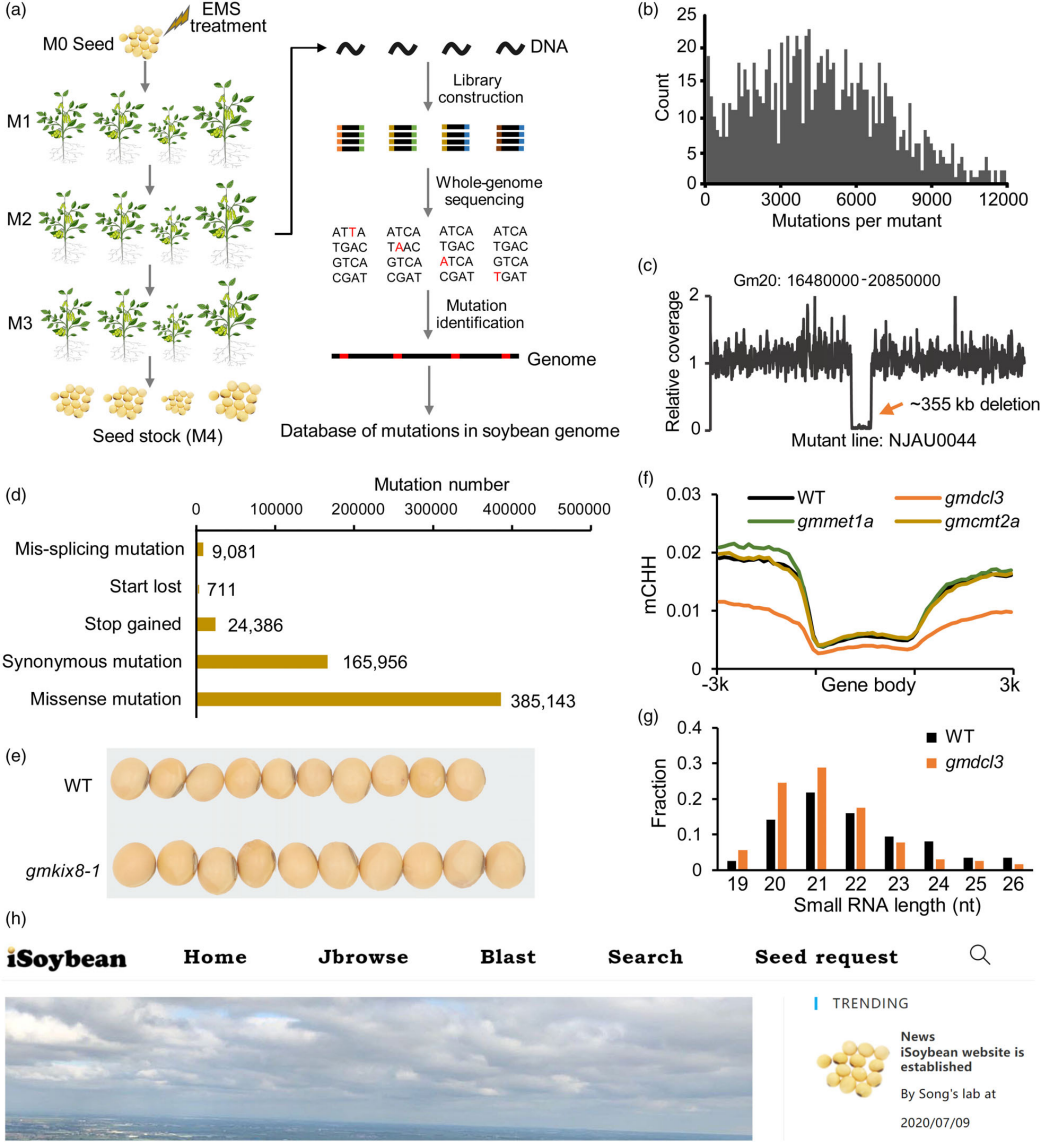
(a) Soybean seeds were subjected to EMS treatment. A single M2 plant for each mutant line was used for construction of WGS library and harvest of M3 seeds. The M3 seeds were planted to obtain M4 seeds for seed stock. Nucleotides in red indicate identified mutations. (b) Distribution of number of mutations per mutant line. (c) Example of a large deletion in mutant line NJAU0044. (d) Functional annotation of mutations located in coding sequences of soybean genes. (e) Knockout of *GmKIX8‐1* (NJAU1840, stop gained) induced larger seeds. (f) CHH methylation changes in gene region in *gmdcl3*, *gmmet1a* and *gmcmt2a* mutants compared with wild type. (g) Size distribution profiles for small RNAs derived from leaves of *gmdcl3* mutant and wild type. (h) The snapshot of the iSoybean website. [Colour figure can be viewed at wileyonlinelibrary.com]

In addition to point mutations, we identified 22 373 small Indels (<50 bp), representing an average of 21.2 small Indels per mutant line (Figure S2a). A total of 1018 genes were found to be affected by 1034 small Indels. Compared with point mutations, small Indels were relatively rare in the mutant population (Figure S2b). Previous studies confirmed EMS mutagenesis could induce large structural variations in rice and wheat (Henry *et al.,* 2014). Through the calculation of coverage variation along chromosomes, we detected 37 large deletions (>20 kb) in 33 mutant lines (Figure 1c). Totally, 401 genes were knocked out by these large deletions (Table S3).

To further analyse the effect of mutations on gene functions, we classified the mutations in gene models into truncation mutations (stop gained, start loss and mis‐splicing), missense mutations and synonymous mutations (Figure 1d). We identified 34 178 truncation mutations, affecting 22 092 protein‐coding genes which account for 41.8% of all soybean genes in reference genome (Figure 1d). In addition, there were 87% (48 613 genes) of soybean genes affected by 385 142 missense mutations. In total, 92.9% of soybean genes were affected by truncation or missense mutation, of which 85% of soybean genes contained two or more non‐synonymous mutations (Figure S3). For example, we observed larger seeds by knockout of *GmKIX8‐1* in mutant NJAU1840 and early flowering due to knockout of *GmE1* in mutant NJAU0143 as reported in previous studies (Figure 1e, Figure S4) (Nguyen *et al.,* 2021; Xia *et al.,* 2012).

The high density of mutations in the gene regions could facilitate functional genomics through forward and reverse genetic approaches. As an example, we examined DNA methylation changes by mutations in genes involved in DNA methylation (Figure S5). In plants, DNA methylation is catalysed in CG, CHG and CHH contexts through maintenance and *de novo* pathways (Figure S5). All homologous genes contained at least one truncation or missense mutation in our mutant population (Figure S5). To examine the effects of these mutations on DNA methylation, we analysed genome‐wide DNA methylation changes by truncation mutations of *GmDCL3* (*Glyma.04G057400*), *GmMET1a* (*Glyma.04G187600*) and *GmCMT2a* (*Glyma.16G103500*), compared with wild type (WT) (Figure S6). No obvious DNA methylation changes in gene region were observed in *gmmet1a* and *gmcmt2a* mutants compared with WT (Figure 1f), which may be due to gene redundancy of *GmMET1* and *GmCMT2* in soybean genome (Figure S5). There is only one homologue of *Arabidopsis DCL3* gene in soybean. Expectedly, *gmdcl3* mutant showed much lower CHH methylation levels in the gene region than WT (Figure 1f). Consistent with the function of *DCL3* in the generation of 24‐nt small RNAs (smRNAs), small RNA‐seq analysis revealed a substantial decrease of 24‐nt smRNAs in *gmdcl3* mutant compared with WT (Figure 1g). These results demonstrate the feasibility of this mutant population to elucidate gene function through reverse genetics.

To make the mutant population available to soybean researchers, we established a website named iSoybean (www.isoybean.org) (Figure 1h). Users can search for mutations for a specific gene or browse all mutations in genomes using a JBrowse graphic interface. The desired mutant seeds can be freely requested from the Nanjing Agricultural University using iSoybean website. In conclusion, our sequenced mutant population provides valuable open‐access resource for mutation discovery and will facilitate functional genomic studies to promote genetic breeding in soybean.

## Conflict of interest

The authors declare no competing interests.

## Authors’ contributions

Q.S. conceived and designed the research. M.Z., X.Z., L.Q., G.J. and W.Y. performed the experiments. M.Z., X.J., L.W. and Q.S. analysed the data. Q.S. wrote the manuscript.

## Supporting information


**Figure S1** Phenotypic variation of soybean mutations. The variations of plant architecture (a) and seed (b) in soybean M3 mutants.
**Figure S2** Distribution of small Indels in the soybean mutant population. (a) Distribution of small Indels per mutant line in the mutant population. (b) Frequency of mutant lines carrying different numbers of small Indels in the gene region.
**Figure S3** Frequency of genes carrying different numbers of non‐synonymous mutations, including stop gained, mis‐splicing, start lost and missense mutations.
**Figure S4** Knockout of GmKIX8‐1 and GmE1 genes in the mutant population. (a) Genomic location and amino acid change of homozygous EMS‐induced mutation in gmkix8‐1 mutant (NJAU1840). (b) Genomic location and amino acid change of homozygous EMS‐induced mutation in gme1 mutant (NJAU0143). (c) Phenotype of wild type (WT) and gme1 mutant. WT did not bloom when gme1 mutant was flowering in long‐day condition (16 h/8 h, light/dark). (d) Flowering time of WT and gme1 mutant in long‐day condition (16 h/8 h, light/dark). DAE: days after emergence.
**Figure S5** Mutations identified in genes involved in DNA methylation and demethylation. Genes in red indicate genes carrying truncation mutations, including stop gained, mis‐splicing and start lost. Genes in brown indicate genes carrying missense mutations.
**Figure S6** Validation of truncation mutations in DNA methylation‐related genes. (a–c) Sanger sequencing validation of truncation mutations in GmMET1a (a), GmCMT2a (b) and GmDCL3 (c) respectively. Black triangles indicate positions of mutations on genes. The retained intron in mature mRNA in gmdcl3 mutant is indicated in blue.

**Appendix S1** Supplementary methods.

**Table S1** Mutations in genome and coding sequence per mutant line.

**Table S2** Mutation validation by sanger sequencing. (Mutation in red indicates false positive of mutation identification).

**Table S3** Genes located in large deletion.

## Data Availability

The data reported in this study have been deposited in the Genome Sequence Archive in the BIG Data Center (https://bigd.big.ac.cn/gsa/index.jsp) under Accession Number PRJCA003200.
