# Supplementary material for: iSoybean: A database for the mutational fingerprints of soybean
Source: Plant Biotechnol J. 2022 Jun 11;20(8):1435–7. doi: 10.1111/pbi.13844 (PMC9342614; doi:10.1111/pbi.13844)
Supplement: Supplementary file 1 — Figure S1 Phenotypic variation of soybean mutations. The variations of plant architecture (a) and seed (b) in soybean M3 mutants. Figure S2 Distribution of small Indels in the soybean mutant population. (a) Distribution of small Indels per mutant line in the mutant population. (b) Frequency of mutant lines carrying different numbers of small Indels in the gene region. Figure S3 Frequency of genes carrying different numbers of non‐synonymous mutations, including stop gained, mis‐splicing, start lost and missense mutations. Figure S4 Knockout of GmKIX8‐1 and GmE1 genes in the mutant population. (a) Genomic location and amino acid change of homozygous EMS‐induced mutation in gmkix8‐1 mutant (NJAU1840). (b) Genomic location and amino acid change of homozygous EMS‐induced mutation in gme1 mutant (NJAU0143). (c) Phenotype of wild type (WT) and gme1 mutant. WT did not bloom when gme1 mutant was flowering in long‐day condition (16 h/8 h, light/dark). (d) Flowering time of WT and gme1 mutant in long‐day condition (16 h/8 h, light/dark). DAE: days after emergence. Figure S5 Mutations identified in genes involved in DNA methylation and demethylation. Genes in red indicate genes carrying truncation mutations, including stop gained, mis‐splicing and start lost. Genes in brown indicate genes carrying missense mutations. Figure S6 Validation of truncation mutations in DNA methylation‐related genes. (a–c) Sanger sequencing validation of truncation mutations in GmMET1a (a), GmCMT2a (b) and GmDCL3 (c) respectively. Black triangles indicate positions of mutations on genes. The retained intron in mature mRNA in gmdcl3 mutant is indicated in blue. [file PBI-20-1435-s002.pdf]

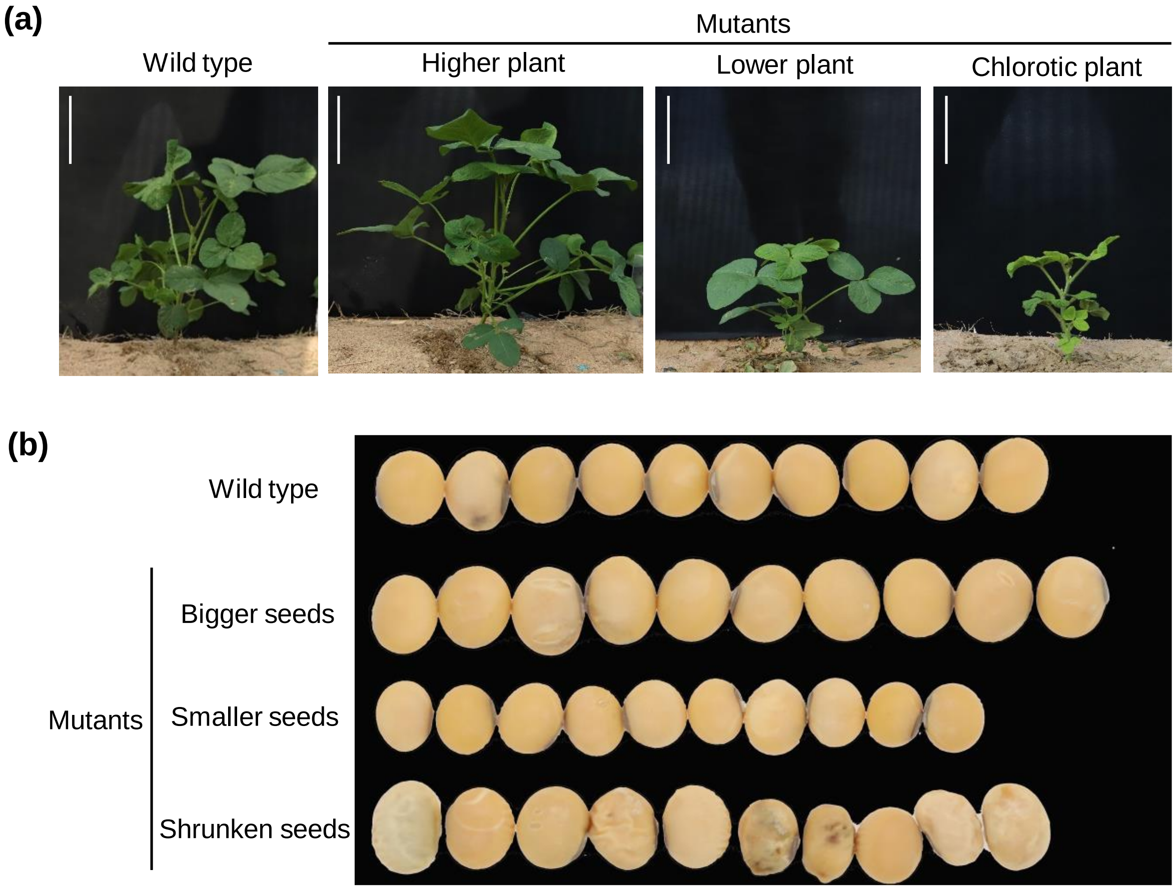

Figure S1. Phenotypic variation of soybean mutations. The variations of plant architecture (**a**) and seed (**b**) in soybean M3 mutants

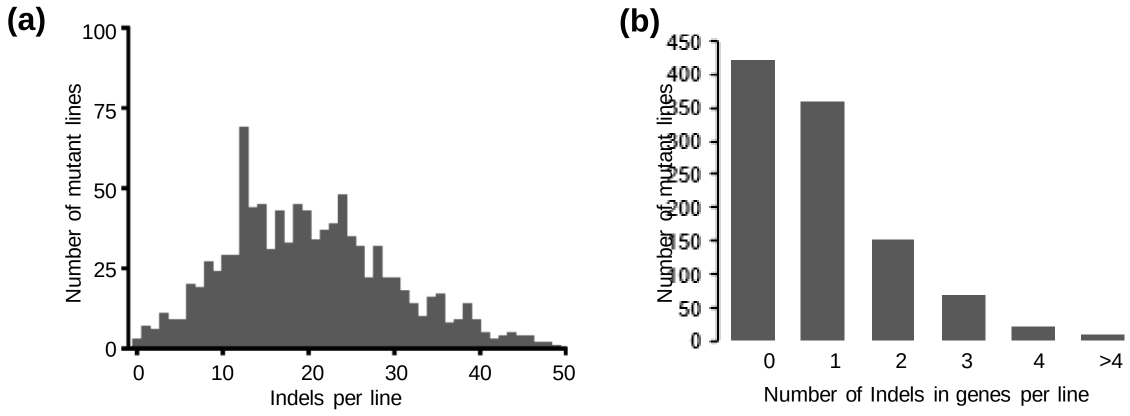

Figure S2. Distribution of small Indels in soybean mutant population.

(a) Distribution of small Indels per mutant line in mutant population.

(b) Frequency of mutant lines carrying different number of small Indels in gene region.

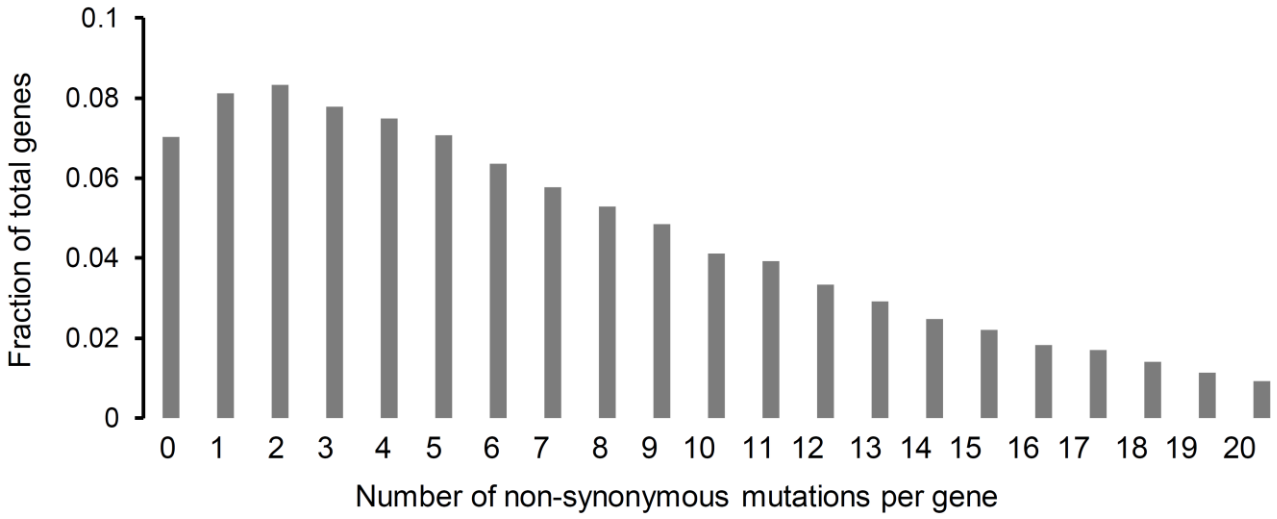

Figure S3. Frequency of genes carrying different number of non-synonymous mutations, including stop gained, mis-splicing, start lost, and missense mutations.

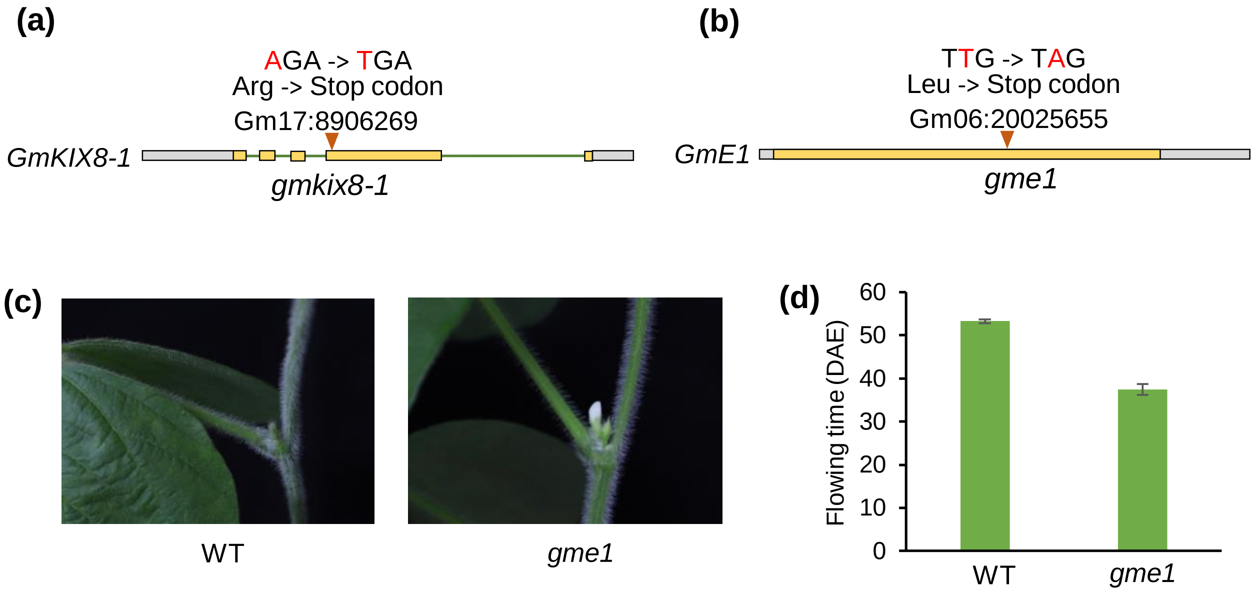

Figure S4. Knockout of *GmKIX8-1* and *GmE1* genes in mutant population.

(a) Genomic location and amino acid change of homozygous EMS-induced mutation in *gmkix8-1* mutant (NJAU1840).

(b) Genomic location and amino acid change of homozygous EMS-induced mutation in *gme1* mutant (NJAU0143).

(c) Phenotype of wild type (WT) and *gme1* mutant. WT did not bloom when *gme1* mutant was flowering in long-day condition (16 h/8 h, light/dark).

(d) Flowering time of WT and *gme1* mutant in long-day condition (16 h/8 h, light/dark). DAE: days after emergence.

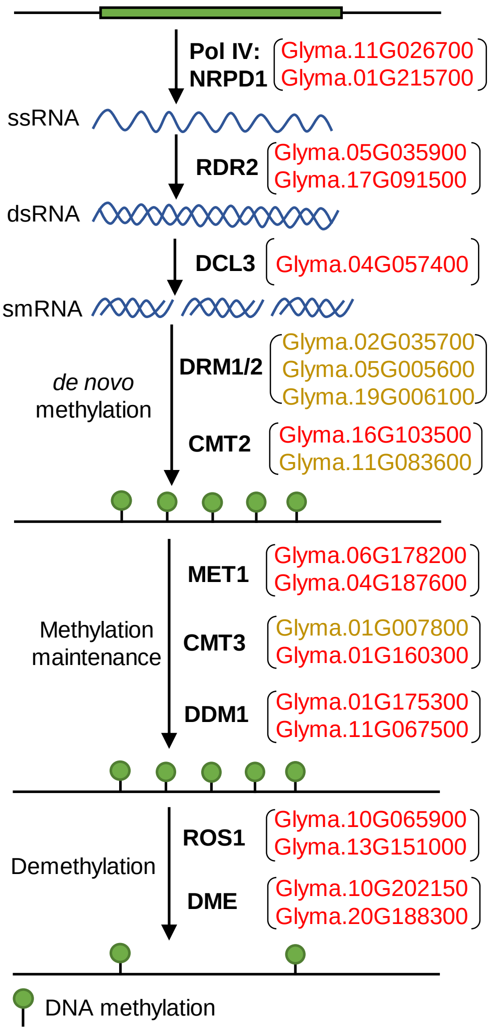

Figure S5. Mutations identified in genes involved in DNA methylation and demethylation. Genes in Red indicate genes carrying truncation mutations, including stop gained, mis-splicing, start lost. Genes in brown indicates genes carrying missense mutations.

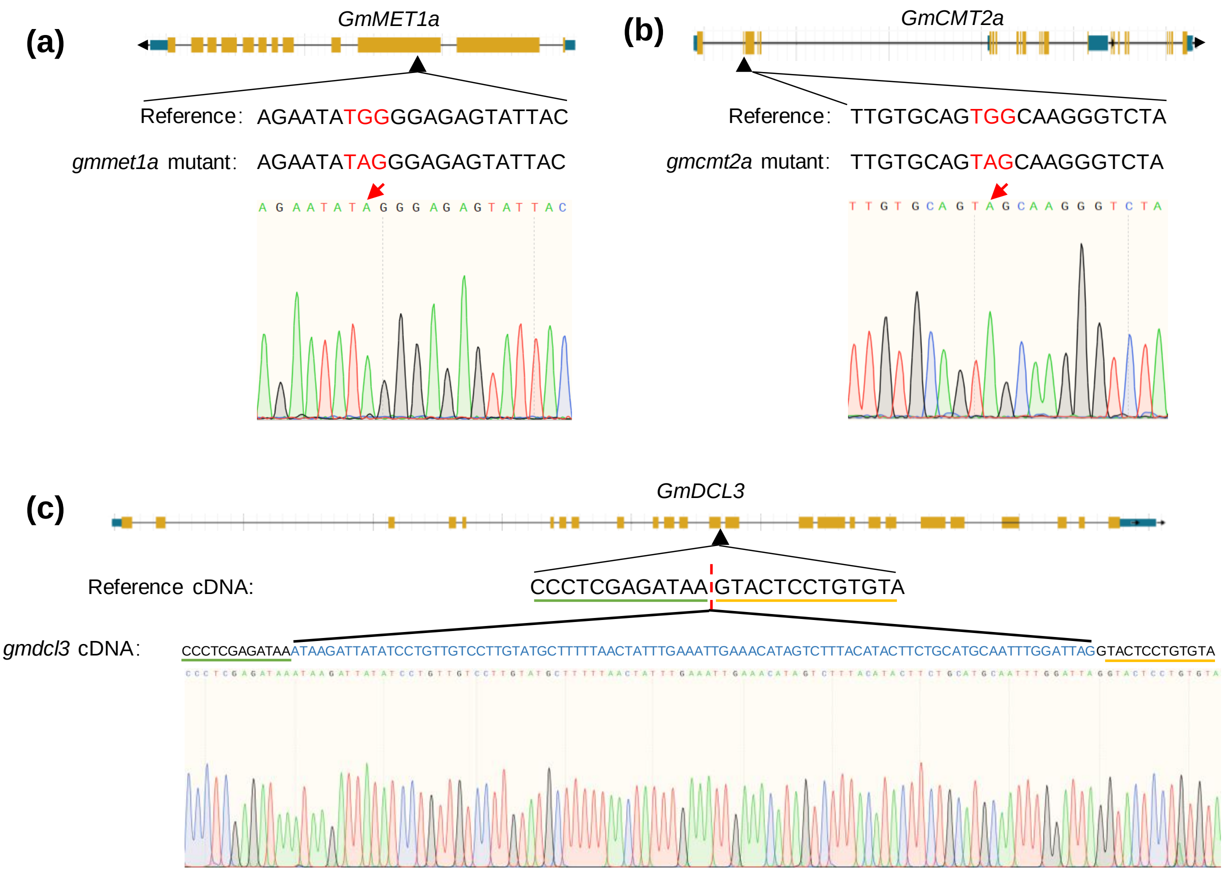

Figure S6. Validation of truncation mutations in DNA methylation related genes. **(a-c)** Sanger sequencing validation of truncation mutations in *GmMET1a* **(a)**, *GmCMT2a* **(b)** and *GmDCL3* **(c)**, respectively. Black triangles indicate positions of mutations on genes. Retained intron in mature mRNA in *gmdcl3* mutant is indicated in blue.
