# Supplementary material for: iSoybean: A database for the mutational fingerprints of soybean
Source: Plant Biotechnol J. 2022 Jun 11;20(8):1435–7. doi: 10.1111/pbi.13844 (PMC9342614; doi:10.1111/pbi.13844)
Supplement: Supplementary file 2 — Appendix S1 Supplementary methods. [file PBI-20-1435-s004.docx]

Supplemental Methods for

**iSoybean: a database for the mutational fingerprints of soybean**

**Plant Material and EMS Mutagenesis**

Mutagenesis was performed as previously described (Li et al., 2017). In brief, approximately 4,000 seeds of Glycine max variety Williams 82 were subjected to 50 mM EMS treatment for 10 hours and then washed with running tap water overnight. The M1 seeds were planted in the field without soybean planting for at least five years at experimental station of Jiangsu Academy of Agricultural Sciences at Nanjing. The M2 seeds were harvested from 1,044 surviving M1 plants and then planted in the experimental station of Nanjing Agricultural University at Hainan. The M3 seeds were harvested from only one surviving plant of each M2 mutant, wherein leaves were harvested for DNA extraction and construction of WGS library. About twenty M3 seeds of each line were planted and the M4 seeds were harvested from all M3 plants for seed stock.

For MethylC-seq, soybean plants were grown in green house under the light/dark (L/D) cycle of 16 h/L and 8 h/D at 25 °C.

**DNA Extraction and Construction of WGS Libraries**

The genomic DNA from M2 plants was extracted using cetyltrimethylammonium bromide (CTAB) based method. About 1μg of genomic DNA was fragmented to 100–1000 bp using Bioruptor (Diagenode). WGS libraries were constructed using NEBNext® Ultra™ II DNA Library Prep Kit (NEB) according to the manufacturer’s instructions. WGS libraries were sequenced on NovaSeq platform (Illumina) for 150 bp paired-end reads.

**Read Mapping of WGS and Identification of Point Mutations and Small Indels**

After adapter clipping and quality filter using Trimmomatic, reads were mapped to genome sequence of *Glycine max* var. Williams 82 (Wm82v4 version) using BWA software with default parameters (Li and Durbin, 2009). Only uniquely mapped reads were kept for further analysis. Alignments were sorted using SAMtools and PCR duplicates were removed using Picard(Li et al., 2009). SNP was first identified using BCFtools and then SNPs were removed if found in two or more sequenced lines (parental line and mutant lines) (Li, 2011). The filtered SNPs were used for base quality recalibration to correct for biases in the quality scores assigned by the sequencer using Genome Analysis Toolkit (GATK) (McKenna et al., 2010). Then, SNP calling was performed using GATK to generate raw point mutations and small Indels dataset for each line. Raw point mutations and small Indels were further filtered with following criteria: 1) phred-scaled quality score was higher than 60; 2) point mutations or small Indel was only found in one sequenced line; 3) heterozygous and homozygous mutations were respectively covered by at least 5 and 3 reads; 4) heterozygosity value was above 0.4 for each heterozygous mutations.

**Identification of Large Deletions**

Large deletions were identified as previously described (Henry et al., 2014). In brief, the soybean reference genome was divided into 5-kb non-overlapping bins. For each bin in each sample, the percentage of reads mapping to that particular bin was recorded. To reduce the mapping bias, percentages per bin were normalized to the mean percentage of reads mapping to that bin for all samples. A regions showing normalized coverage of at least four adjacent bins with values below 0.5 indicates the presence of a large deletion.

**Validation of EMS Mutations**

A total of 105 GC>AT and 45 nonGC>AT mutations were randomly selected for validation. The 300 bp flanking sequences of mutations were extracted for primer design. PCR was performed with primers listed in Table S2 and amplified products were subjected to Sanger sequencing.

**Construction of MethylC-seq Libraries and Read Mapping**

MethylC-seq libraries were constructed with two biological replicates for *gmdcl3* and one biological replicate for wild type, *gmmet1a* and *gmcmt2a* mutants as described (Song et al., 2017). In brief, genomic DNA was isolated from first trifoliate leaves at three weeks after planting using CTAB method. About 1μg of genomic DNA was fragmented to 100–1000 bp using Bioruptor (Diagenode). End repair was performed on the DNA fragment using NEBNext® End Repair Module (NEB), followed by adding an 'A' base to the 3'end using NEBNext® dA-Tailing Module (NEB). The resulting DNA fragment was ligated to the methylated DNA adapter using Quick Ligation™ Kit (NEB). The adapter-ligated DNA of 200-400 bp was purified using AMPure beads, followed by sodium bisulfite conversion using EZ DNA Methylation-Gold Kits (ZYMO RESEARCH). The bisulfite-converted DNA was amplified by 12 cycles of PCR using KAPA HiFi HotStart Uracil+ ReadyMix Kit (Roche). MethylC-seq libraries were sequenced on NovaSeq platform (Illumina) for 150 bp paired-end reads.

MethylC-seq reads were mapped to genome sequence of *Glycine max* var. Williams 82 (Wm82v4 version) using Bismark with parameters (--score_min L,0,-0.2 -X 1000 --no-mixed --no-discordant) (Krueger and Andrews, 2011). Only reads mapped to the unique sites were retained and used for further analysis. The reads mapped to the same site were collapsed into a single consensus read to reduce clonal bias.

**Construction of Small RNA-seq Libraries and Read Mapping**

Total RNA was isolated from first trifoliate leaves at three weeks after planting using Trizol Reagent (Life Technologies). Small RNA-seq libraries were constructed using NEBNext® Small RNA Library Prep Set (NEB) according to the manufacturer’s instructions and then sequenced on HiSeq 2500 platform (Illumina) for 50 bp single-end reads.

After adapter clipping, small RNA-seq reads were mapped to genome sequences of *Glycine max* (Williams 82) using Bowtie settings with parameters (-S -q -a -v 0 -p 4) (Langmead et al., 2009). Multi-mapped small RNA-seq reads (<50 mapping locations) were weighted and annotated using ShortStack software with parameters (--bowtie_cores 16 --nohp --pad 100 --bowtie_m 50 --align_only) (Axtell, 2013).

**References**

Axtell, M.J. (2013) ShortStack: comprehensive annotation and quantification of small RNA genes. *RNA* **19**, 740-751.

Henry, I.M., Nagalakshmi, U., Lieberman, M.C., Ngo, K.J., Krasileva, K.V., Vasquez-Gross, H., Akhunova, A., Akhunov, E., Dubcovsky, J., Tai, T.H. and Comai, L. (2014) Efficient Genome-Wide Detection and Cataloging of EMS-Induced Mutations Using Exome Capture and Next-Generation Sequencing. *Plant Cell* **26**, 1382-1397.

Krueger, F. and Andrews, S.R. (2011) Bismark: a flexible aligner and methylation caller for Bisulfite-Seq applications. *Bioinformatics* **27**, 1571-1572.

Langmead, B., Trapnell, C., Pop, M. and Salzberg, S.L. (2009) Ultrafast and memory-efficient alignment of short DNA sequences to the human genome. *Genome Biol* **10**, R25.

Li, H. (2011) A statistical framework for SNP calling, mutation discovery, association mapping and population genetical parameter estimation from sequencing data. *Bioinformatics* **27**, 2987-2993.

Li, H. and Durbin, R. (2009) Fast and accurate short read alignment with Burrows-Wheeler transform. *Bioinformatics* **25**, 1754-1760.

Li, H., Handsaker, B., Wysoker, A., Fennell, T., Ruan, J., Homer, N., Marth, G., Abecasis, G., Durbin, R. and Genome Project Data Processing, S. (2009) The Sequence Alignment/Map format and SAMtools. *Bioinformatics* **25**, 2078-2079.

Li, Z., Jiang, L., Ma, Y., Wei, Z., Hong, H., Liu, Z., Lei, J., Liu, Y., Guan, R., Guo, Y., Jin, L., Zhang, L., Li, Y., Ren, Y., He, W., Liu, M., Htwe, N.M., Liu, L., Guo, B., Song, J., Tan, B., Liu, G., Li, M., Zhang, X., Liu, B., Shi, X., Han, S., Hua, S., Zhou, F., Yu, L., Li, Y., Wang, S., Wang, J., Chang, R. and Qiu, L. (2017) Development and utilization of a new chemically-induced soybean library with a high mutation density. *J Integr Plant Biol* **59**, 60-74.

McKenna, A., Hanna, M., Banks, E., Sivachenko, A., Cibulskis, K., Kernytsky, A., Garimella, K., Altshuler, D., Gabriel, S., Daly, M. and DePristo, M.A. (2010) The Genome Analysis Toolkit: a MapReduce framework for analyzing next-generation DNA sequencing data. *Genome Res* **20**, 1297-1303.

Song, Q., Zhang, T., Stelly, D.M. and Chen, Z.J. (2017) Epigenomic and functional analyses reveal roles of epialleles in the loss of photoperiod sensitivity during domestication of allotetraploid cottons. *Genome Biol* **18**, 99.
